# Osteoclastogenesis inhibition by mutated IGSF23 results in human osteopetrosis

**DOI:** 10.1111/cpr.12693

**Published:** 2019-09-27

**Authors:** Ying Yuan, Li Yang, Ting Liu, Hong Zhang, Qiong Lu

**Affiliations:** ^1^ Institute of Endocrinology and Metabolism The Second Xiangya Hospital Central South University Changsha Hunan China; ^2^ Department of Endocrinology Hunan Provincial People's Hospital Changsha Hunan China; ^3^ Department of Pharmacy The Second Xiangya Hospital Central South University Changsha Hunan China

**Keywords:** IGSF23, mutation, osteopetrosis, peripheral blood mononuclear cells

## Abstract

**Objectives:**

Osteopetrosis is a rare inherited skeletal disease characterized by increased bone mineral density due to the loss of osteoclast function or differentiation potential.

**Materials and Methods:**

The study involved a Chinese patient with osteopetrosis (the proband) and her immediate family members and 180 controls without osteopetrosis. Bone density of the femoral neck, lumbar spine and total body was measured using dual‐energy x‐ray absorptiometry. Osteoclast differentiation by the participants’ peripheral blood mononuclear cells (PBMCs) was investigated using tartrate‐resistant acid phosphatase (TRAP) staining. Osteoblast differentiation was examined with Alizarin Red S staining. Reverse transcription‐quantitative PCR was used to amplify immunoglobulin superfamily member 23 (*IGSF23*), c‐*FOS* and nuclear factor of activated T cells 1 (NFATC1).

**Results:**

We found a homozygous mutation (c.295C>T) in the *IGSF23* gene in two osteopetrosis samples. The mutation led to the formation of a stop codon, causing loss of the immunoglobulin‐like domain and the whole transmembrane domain. PBMCs from the proband (*IGSF23* ^−/−^) exhibited poor ability for differentiating into mature osteoclasts in vitro. Overexpression of *IGSF23* rescued the ability of *IGSF23* ^−/−^ PBMCs to differentiate into osteoclasts. Moreover, knockdown of IGSF23 reversed the bone loss in OVX mice by injecting AAV‐shIGSF23 into mice femoral bone marrow cavity. Furthermore, we also found that the *IGSF23* mutation led to decreased c‐Fos and NFATC1 expression levels by inhibiting the mitogen‐activated protein kinase signalling pathways.

**Conclusions:**

IGSF23‐mediated osteoclast differentiation of PBMCs may serve as a potential target in osteoporosis therapy.

## 1 INTRODUCTION

Bone homeostasis tightly depends on the balance between bone resorption and bone formation.^1^, ^2^, ^3^, ^4^ A perturbation of this balance will lead to some bone metabolic diseases including osteoporosis and osteopetrosis.^5^, ^6^ Especially, osteopetrosis is regarded as a kind of rare inherited skeleton disease characterized by increased bone mineral density and lost of osteoclast function or differentiation potential.^7^, ^8^, ^9^

In humans, osteopetrosis is classified into autosomal recessive and autosomal dominant types on the basis of mode of inheritance. To date, at least 12 genes mutation including TCIRG1, CLCN7, LRP5, IGSF23, OSTM1, CAII, PLEKHM1, TNFSF11, TNFRSF11A, CTSK, IKBKG and ITGB3 have been reported to associated with human osteopetrosis.^10^, ^11^, ^12^, ^13^ Most of these mutations cause osteoclast‐rich osteosclerosis characterized by increasing the amount of osteoclasts but the activity of bone resorption is weakened or lost.^11^ However, the mutation of RANKL (TNFSF11) or its receptor RANK (TNFRSF11A) cause a kind of more rarely osteopetrosis characterized by lacking of mature osteoclasts.^14^, ^15^

In this study, we found a novel pathogenic gene mutation (IGSF23) from an autosomal recessive osteopetrosis pedigree, the mutation of IGSF23 lead to osteopetrosis as a result of preventing osteoclastogenesis of PBMCs.^16^

## 2 MATERIALS AND METHODS

### 2.1 Patients

**Figure 1:**
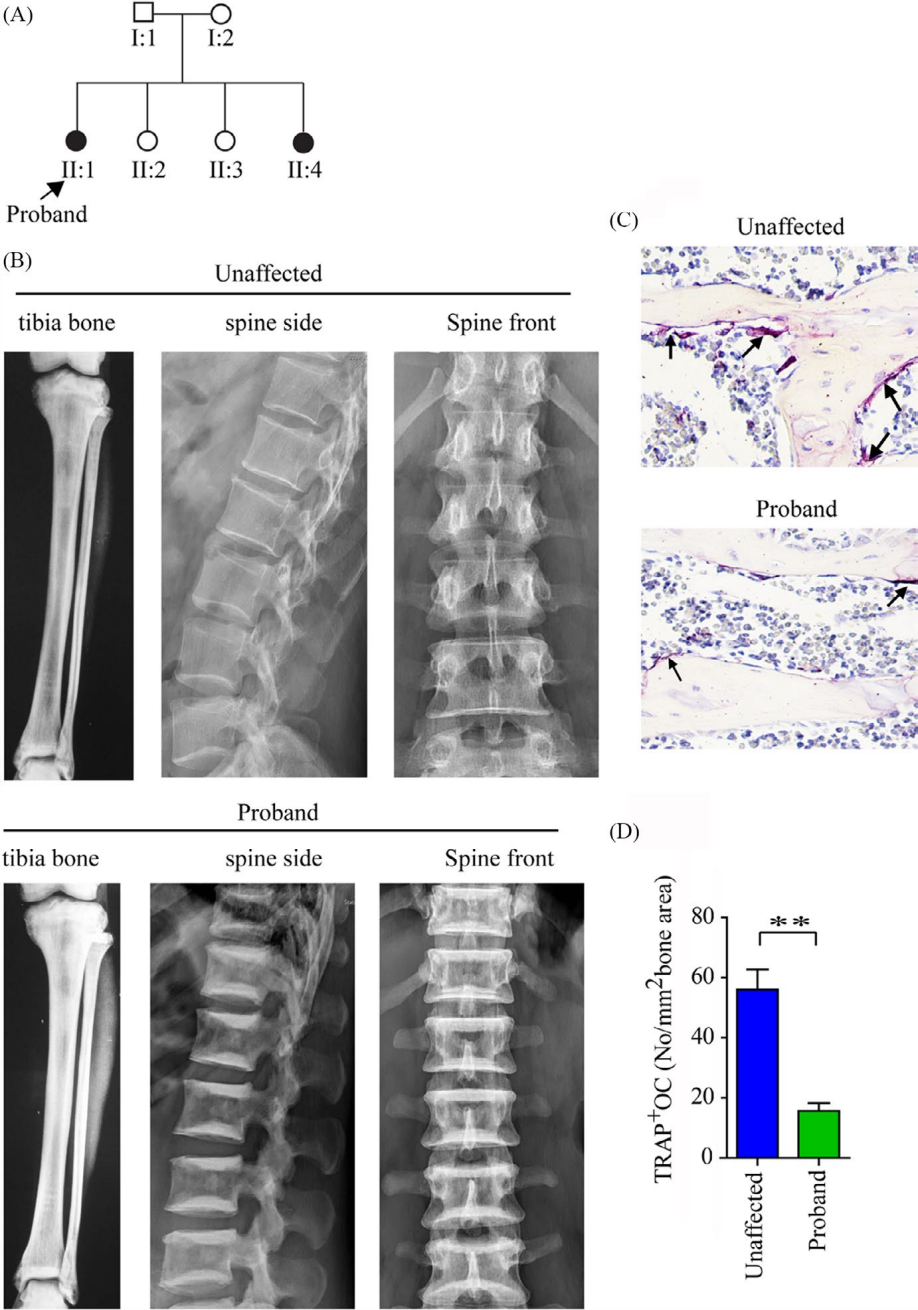
The characteristic of the Chinese autosomal recessive osteopetrosis family. A, Affected family members are indicated by black symbols. B, X‐rays showed the characteristic of tibia bone and spine from proband (II:1) and unaffected family members. C, Osteoclasts stained by TRAP staining analysis from the proband and unaffected family members, arrows indicate osteoclasts (TRAP‐positive). D, Quantitative analysis of TRAP staining analysis. **P < .01

The family members (Figure 1) and 180 normal controls involved in this study were recruited by Second Xiangya Hospital. All subjects were Han ethnicity and were recruited from a local population of Hunan Province located in central south China.

### 2.2 Cells culture and vectors

Peripheral blood mononuclear cells were isolated and purified from blood samples as previous described.^17^ Briefly, the 25 mL diluted blood samples was added with 12 mL Ficoll^®^ PM 400 Histopaque^®^‐1077 (Sigma), and then centrifugated at 1000 *g* for 10 minutes. After centrifugation, PBMCs was adsorbed with a transpirator and seeded on 10‐cm tissue culture dish with 15 mL α‐MEM (Gibco) containing 10% foetal bovine serum (FBS) and 1% streptomycin and penicillin. Primary osteoclasts were isolated from human vertebral cancellous bone as previous described.^18^

Osteoblasts (passage 3) were regarded as a gift from Department of Endocrinology Research Center of Xiangya Hospital. Osteoclasts and osteoblasts were cultured in α‐MEM complete medium at 37°C and 5% CO_2_ incubator (Thermo Fisher Scientific, Inc).

The full‐length IGSF23 cDNA was generated by PCR with Forward primer 5′ GAATTCCGACCACCACCACTGACCCG 3′ and Reverse primer 5′ CGGGATCCTCAGCTGCATATTCCTA 3′. The PCR product was digested with EcoR I and BamHI fast restriction endonuclease and constructed into pHBLV‐CMV‐IRES‐Puro vector (Hanbio Biotechnology). The lentivirus produced IGSF23‐shRNAs were purchased from Hanbio Biotechnology (Shanghai) and the target sequences for IGSF23‐shRNAs are as follows: 5′‐CTGCTATCCGGGGAGTCATC‐3′; 5′‐TCATGCAGCCCACAGAAGCAG‐3′.

### 2.3 Bone density measurements

The femoral neck, lumbar spine and total body bone density were measured by dual‐energy x‐ray absorptiometry. The results are expressed as *Z*‐scores.^19^

### 2.4 Mutational analysis

Peripheral blood was collected from all family members and normal controls, and the blood genomic DNA was extracted with FlexiGene^@^ DNA kit (Qiagen).^20^ The exons of candidate genes for osteopetrosis were amplified and sequenced by DNA sequencing instrument (llumine 2500). Specific primers for amplification of IGSF23 exons are provided in Table S1.

### 2.5 Protein fractionation

The PBMCs were collected after treated with M‐CSF and RANKL for 3 days. Cells were fractionated into cytosol, membrane and nuclei using Qproteome cell compartment kit (Qiagen) according to the manufacturer's instructions. And then, the cytosol, membrane and nuclei fraction were subjected to Western blotting analysis.

### 2.6 Osteoclast differentiation and TRAP staining

Peripheral blood mononuclear cells were cultured and seeded into 48‐well plates with glass coverslips containing 10% FBS, 50 ng/mL M‐CSF (R&D) and 100 ng/mL RANKL (R&D). Cells were treated for 7 days, osteoclasts containing more than three nuclei were generated and further identified by tartrate‐resistant acid phosphatase (TRAP) staining.^21^ TRAP staining was performed as previous.^22^ Images were captured with a Diaphot Inverted Microscope and Camera System (Leica). TRAP‐positive multinuclear cells were observed and counted in 5 random regions per sample.^23^

### 2.7 Osteoblast differentiation and Alizarin Red S Staining

Osteoblasts were culture with osteogenesis induction medium containing 10% FBS, 300 ng/mL BMP‐2, 50 μg/mL ascorbic acid and 5 mmol/L β‐glycerol phosphate for 21 days. After 21 days, cells were fixed by paraformaldehyde (4%) for 10 minutes. And then, cells were stained with 2% Alizarin Red S staining (Sigma). The cell matrix mineralization was imaged by a Camera System (Leica), n = 5 per group.^24^, ^25^, ^26^

### 2.8 RT‐qPCR analysis

Total RNA of tissues or cells was extracted using Trizol reagent (Invitrogen), and transcribed into cDNA by using SuperScript II Kit (Invitrogen). The primers of IGSF23, c‐Fos, NFATc1 and GAPDH were shown: IGSF23 5′‐CCGATGCTAGAGAAGGATGC‐3′ and 5′‐AAACACATGCCTGCAATCAG‐3′; Cathepsin K 5′‐ ACACCCACTGGGAGCTATG ‐3′ and 5′‐ GACAGGGGTACTTTGAGTCCA ‐3′; MMP9 5′‐ TGTACCGCTATGGTTACACTCG ‐3′ and 5′‐ GGCAGGGACAGTTGCTTCT‐3′; c‐Fos 5′‐ CCGGGGATAGCCTCTCTTACT‐ 3′ and 5′‐ CCAGGTCCGTGCAGAAGTC‐3′; NFATc1 5′‐ CACCGCATCACAGGGAAGAC‐3′ and 5′‐ GCACAGTCAATGACGGCTC‐3′; GAPDH 5′‐GAAGGTGAAGGTCGGAGTCA‐3′ and 5′‐CATTGCTGATGATCTTGAGG‐3′. QRT‐PCR was performed as follows: 94°C for 30 seconds, 60°C for 1 minutes for 40 cycles. The expression level of a target gene calculated using the 2^‐△△Ct^ method. GAPDH used as an internal control.^27^

### 2.9 Western blot analysis

Tissues and cells were collected and lysed with 400 μL RIPA buffer containing 1× protease inhibitors (Roche) on ice for 30 minutes. After 30 minutes, the samples were centrifugated, denaturated and separated by 12% SDS gel electrophoresis as previous.^28^ The rabbit anti‐IGSF23 polyclonal antibody was raised against the linear peptide sequence MQPTEAEPMEPDPTLS of the predicted human IGSF23 protein (NP_001192209), β‐actin, RANK, c‐Fos, NFATc1, p‐ERK, ERK, p38, p‐p38 antibodies were purchased from Cell Signaling Technology; mSin3A (Abcam), 5'NT, D4‐GDI and TRAP antibodies were purchased from Abcam.

### 2.10 Toluidine blue staining

Peripheral blood mononuclear cells were cultured in 24‐well plates with bovine bone slices containing 10% FBS, 50 ng/mL M‐CSF and 100 ng/mL RANKL. After treating for 21 days, the slices were cleaned of cells and stained with 0.1% toluidine blue, and observed by conventional light microscopy. Bone resorption activity was evaluated by measurement of the area of bone resorption pits using image analysis software (Image J).

### 2.11 Statistical analysis

All data were analysed using SPSS 22.0 software (SPSS Inc). The comparison for multiple groups was performed by one‐way ANOVA analysis, and differences were considered significant at *P* < .05.^29^, ^30^, ^31^, ^32^, ^33^

## 3 RESULTS

### 3.1 Clinical examination

The proband (II:1) was a 41‐year‐old woman admitted to hospital with sacroiliac joint pain. Osteopetrosis was diagnosed by X‐ray analysis. The study of pedigree showed that osteopetrosis in two siblings, but another four family members did not exhibit any symptoms of the disease (Figure 1 A). Skeletal radiographic examinations showed that the proband bone density was significantly increasing in the tibia bone and spine compared to her sister (II:2, an age‐matched healthy individual). Besides, her spine appeared with a “sandwich” appearance in the vertebrae (Figure 1B). Moreover, the BMD in the proband femoral neck, lumbar spine and total body was dramatically increased when compared to unaffected family members (Table 1) by dual‐energy x‐ray absorptiometry analysis. But, both of them the growth parameters (height and weight), blood glucose, liver and kidney function were normal and none of them had any history of fractures. And the levels of calcium, phosphate, PTH and 25‐Hydroxyvitamin D were also normal in serum (Table 2). However, the serum bone resorption biomarker TRAP‐5b and bone formation biomarkers BAP were lower in affected samples compared to unaffected family members (Table 2). Furthermore, the amount of osteoclasts was fewer in the proband compared to her sister (II:2) by TRAP staining (Figure 1C,D).

**Table 1:** Clinical phenotypes in the kindred

Member	Age (y)	IGSF23 genotype	Spine (L1‐L4)	Bone density left femur neck *Z*‐score	Total body
Affected					
II:1. Daughter	44	t/t	7.9	6.8	7.0
II:4. Daughter	47	t/t	7.5	6.1	5.5
Unaffected					
I:1. Father	86	c/t	1.2	1.5	1.1
I:2. Mother	81	c/t	1.7	0.8	0.2
II:2. Daughter	53	c/c	1.0	0.6	0.3
II:3. Daughter	50	c/t	1.4	0.7	0.5

Note

Homozygous affected status, heterozygous carrier status and non‐carrier status according to sequence analysis of c.295c>t.

**Table 2:** Serum indexes of mineral metabolism and serum markers of bone turnover in the kindred

Index or marker	Affected		Unaffected				Normal range
	II:1	II:4	I:1	I:2	II:2	II:3	
Calcium	2.66	2.45	2.03	2.11	2.37	2.41	2.1‐2.6 mmol/L
Phosphate	0.97	1.21	1.18	1.05	1.08	1.22	0.8‐1.4 mmol/L
PTH	26	35	56	62	45	39	13‐89 pg/mL
25(OH)D	34	27	17	16	36	33	11‐40 ng/mL
BAP	11	13	62	53	47	52	17‐75 U/L
TRAP‐5b	2	3	14	22	21	24	6‐28 U/L

Note

Normal ranges of PTH, 25(OH)D, BAP and TRAP‐5b were generated from 180 healthy adults (90 women and 90 men; age range: 40‐80).

Abbreviations: 25(OH)D, 25‐Hydroxyvitamin D; BAP, bone alkaline phosphatase; PTH, parathyroid hormone; TRAP‐5b, tartrate‐resistant acid phosphatase‐5b.

### 3.2 Genetic linkage analysis and IGSF23 mutation detection

**Figure 2:**
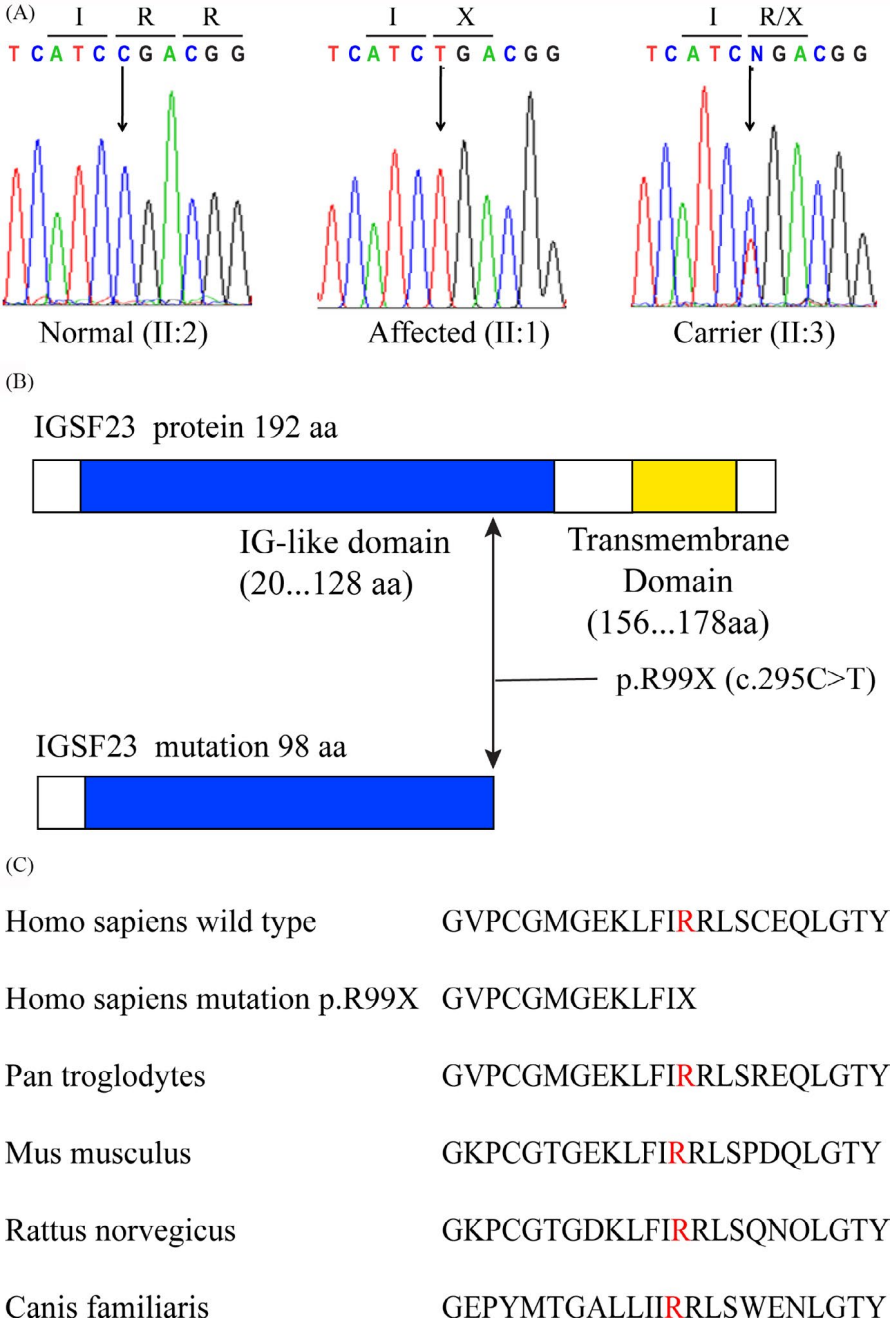
Details of IGSF23 mutation. A, The genomic DNA is performed by sequence analysis from a normal family member (II:2), the proband (II:1) and a heterozygous carrier (II:3), the position of mutation is indicated by arrows. B, The structure pattern of IGSF23 protein (upper) and the mutation IGSF23 (lower). C, The arginine at residue 99 (shown in red) of IGSF23 protein is conserved in different species (NCBI BLAST)

Previous studies demonstrated that 11 genes mutation, including TCIRG1, CLCN7, LRP5, IGSF23, OSTM1, CAII, PLEKHM1, TNFSF11, TNFRSF11A, CTSK, IKBKG and ITGB3 are reported to associate with osteopetrosis. So, we plan to find the genes mutation in the pathogenic family. However, the genes mutation of TCIRG1, CLCN7, LRP5, OSTM1, CAII, PLEKHM1, TNFSF11, TNFRSF11A, CTSK, IKBKG and ITGB3 are not found. Next, the coding region was screened by the high‐throughput sequencing approach. We identified a nonsense mutation (c.295C>T) in exon 2 of IGSF23 (Figure 2A). The mutation of IGSF23 led to the arginine residue (CGA) at codon 99 of IGSF23 protein is substituted by a stop codon TGA (p.R99X; Figure A,B), which predicted to generate a mutated IGSF23 protein with only 98 amino acid residues. Interesting, the arginine residue at the position 99 of IGSF23 protein is highly conserved in mouse, rat, pan troglodytes and canis familiaris (Figure 2C). Lastly, we found that the p.R99X mutation showed a perfect co‐segregation associated with the osteopetrosis phenotype by sequencing of IGSF23 from other five samples. That is, all affected individuals were homozygous mutation, their parents were heterozygous mutation, and two other sisters were either heterozygous mutation (II:3) or normal (II:2). Interestingly, the type of mutation was absent in 180 unrelated normal samples.

### 3.3 IGSF23 expression pattern and subcellular localization

**Figure 3:**
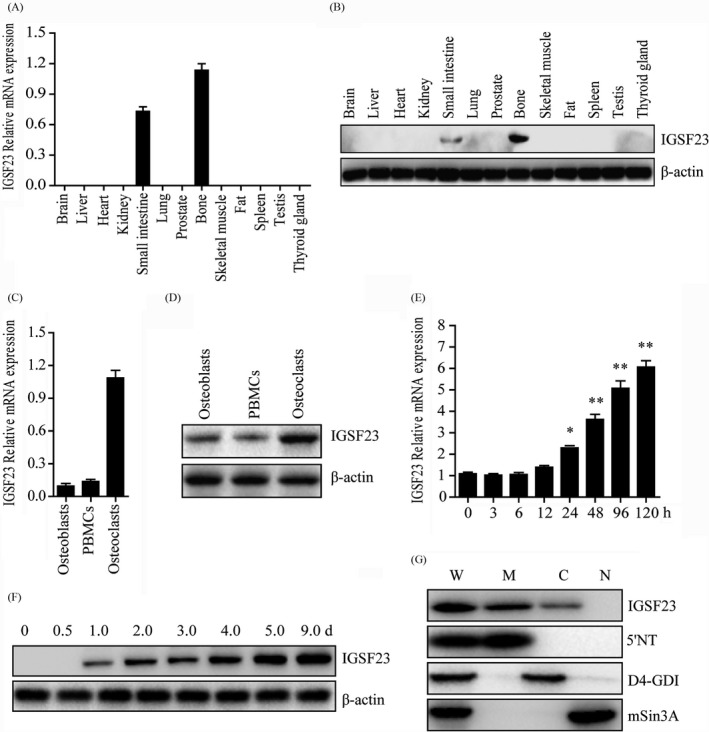
Expression pattern of IGSF23. A, Various adult human tissues were homogenated and isolated the total RNA for qPCR analysis. B, Various adult human tissues were homogenated and lysated for Western blot analysis incubated with IGSF23 antibody. C, Total RNA was isolated from osteoblasts, PBMCs and osteoclasts and was subjected to qPCR analysis. D, Osteoblasts, PBMCs and osteoclasts were collected and lysated for Western blot analysis incubated with IGSF23 antibody. E, PBMCs were collected for qPCR analysis after induced by M‐CSF/RANKL. F, PBMCs were collected for Western blot analysis incubated with IGSF23 antibody. G, PBMCs fractionation were separated and subjected to Western blots analysis incubated with IGSF23 antibody. Three replicates were performed for RT‐qPCR and Western blotting analysis. *P < .05, **P < .01

To confirm IGSF23 gene expression pattern and its subcellular localization, total RNA and protein were isolated from multiple adult human tissues (Clontech Laboratories) are performed. The result of qRT‐PCR and Western blot analysis showed that IGSF23 was especially expressed in bone and small intestine, but was not found in other tissues including brain, liver, heart, kidney, lung, prostate, skeletal muscle, fat, spleen, testis and thyroid gland (Figure A,B). To further understand the expression pattern of IGSF23, the total RNA and protein were isolated from primary human osteoblasts, PBMCs (pre‐osteoclasts) and osteoclasts, qRT‐PCR and Western blot analysis showed that IGSF23 was especially high expressed in osteoclasts, but was low expressed in osteoblasts and PBMCs (Figure 3C,D). Interestingly, the mRNA levels and protein expression of IGSF23 in PBMCs were significantly increased after treating with M‐CSF and RANKL at different time points (Figure 3E,F). To confirm the subcellular localization of IGSF23 protein, the cytoplasmic protein, membrane protein and nucleoprotein were extracted from PBMCs after treated with M‐CSF and RANKL. Western blot analysis showed IGSF23 is mainly expressed in the cell membrane and cytoplasm, but not in the nucleus (Figure 3G). Membrane‐specific antibody (5′NT), cytoplasmic‐specific antibody (D4‐GDI) and anti‐nucleotidase antibodies (mSin3A) were used to confirm there is no contamination between the different fractions. These data demonstrated that IGSF23 expressed in membrane and cytosol, IGSF23 may be involved in the process of osteoclastogenesis.

### 3.4 IGSF23 regulates M‐CSF/RANKL‐induced osteoclastogenesis of PBMCs

**Figure 4:**
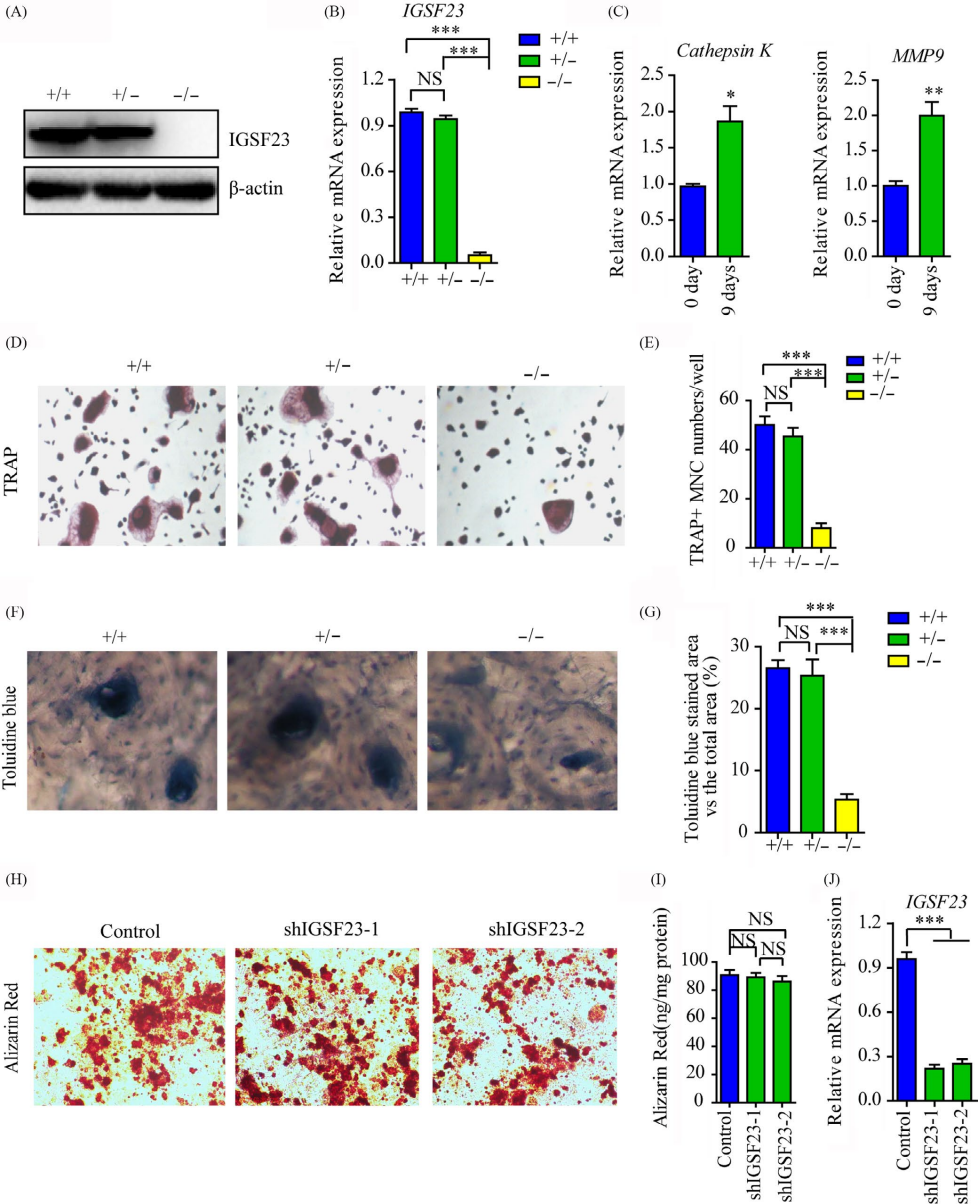
PBMCs from the proband (II:1; −/−) and her two older sisters (II:2; +/+ and II:3; +/−) were seeded into 24‐well plates and induced by M‐CSF/RANKL. A, PBMCs were collected for Western blot analysis incubated with IGSF23 antibody. B, PBMCs were collected for RT‐qPCR analysis. C, RT‐qPCR analysed the mRNA levels of Cathepsin K and MMP9. D, Osteoclasts were imaged and indicated by the arrows (200×). E, The numbers of osteoclast were counted. F, The bone resorption was analysed by toluidine blue staining. G, The percentage bone resorption pit areas were measured. H, Osteoblast cells were transfected with shIGSF23‐1 and shIGSF23‐2 and stained with Alizarin Red staining. I, The matrix mineralization in osteoblasts transfected with shIGSF23‐1 and shIGSF23‐2 was analysed. J, The osteoblasts transfected with shIGSF23‐1 and shIGSF23‐2 and were collected for RT‐qPCR analysis. Three replicates were performed for RT‐qPCR and Western blotting analysis. ***P < .001. NS = Not significant

To investigate whether IGSF23 involved in the process of osteoclastogenesis, PBMCs from the proband (II:1; −/−) and her two older sisters (II:2; +/+ and II:3; +/−) were seeded onto 24‐well plates and incubated with M‐CSF and RANKL for 7 days. Western blot and qRT‐PCR analysis showed that IGSF23 protein was expressed in IGSF23^+/+^ or IGSF23^+/−^ PBMCs, but not in IGSF23^−/−^ after induced by M‐CSF/ RANKL (Figure 4A,B). Moreover, the mRNA levels of Cathepsin K and MMP9 were increased in PBMCs after incubated with M‐CSF and RANKL (Figure 4C). TRAP staining showed that osteoblasts with more than three nuclei/cell and TRAP‐positive (TRAP^+^) were formed in M‐CSF/RANKL‐induced PBMCs from her older sisters II:2 (IGSF23^+/+^) or II:3 (IGSF23^+/−^). However, the number of osteoclasts (TRAP^+^) were much less in M‐CSF/RANKL‐induced PBMCs from the proband (II:1, IGSF23^−/−^) than her older sisters II:2 (IGSF23^+/+^) or II:3 (IGSF23^+/−^) (Figure 4D,E). Toluidine blue staining showed that the percentage bone resorption pit areas was lower in M‐CSF/RANKL‐induced PBMCs from the proband (II:1, IGSF23^−/−^) than her older sisters II:2 (IGSF23^+/+^) or II:3 (IGSF23^+/−^) (Figure 4F,G). These results indicated that IGSF23 is necessary for PBMCs to form osteoclasts. According to our result, we also found that IGSF23 is expressed in osteoblast cells (Figure 3C,D). It suggests that the mutation of IGSF23 maybe affect the function of osteoblasts. To test the hypothesis, we utilized shRNA Lentiviral particles to knockdown IGSF23 in human osteoblasts (Figure 4J). Here, we found that knockdown of IGSF23 without affected osteogenesis by Alizarin Red staining analysis (Figure 4H,I). The result demonstrates that IGSF23 don′t affect the function of osteoblasts.

**Figure 5:**
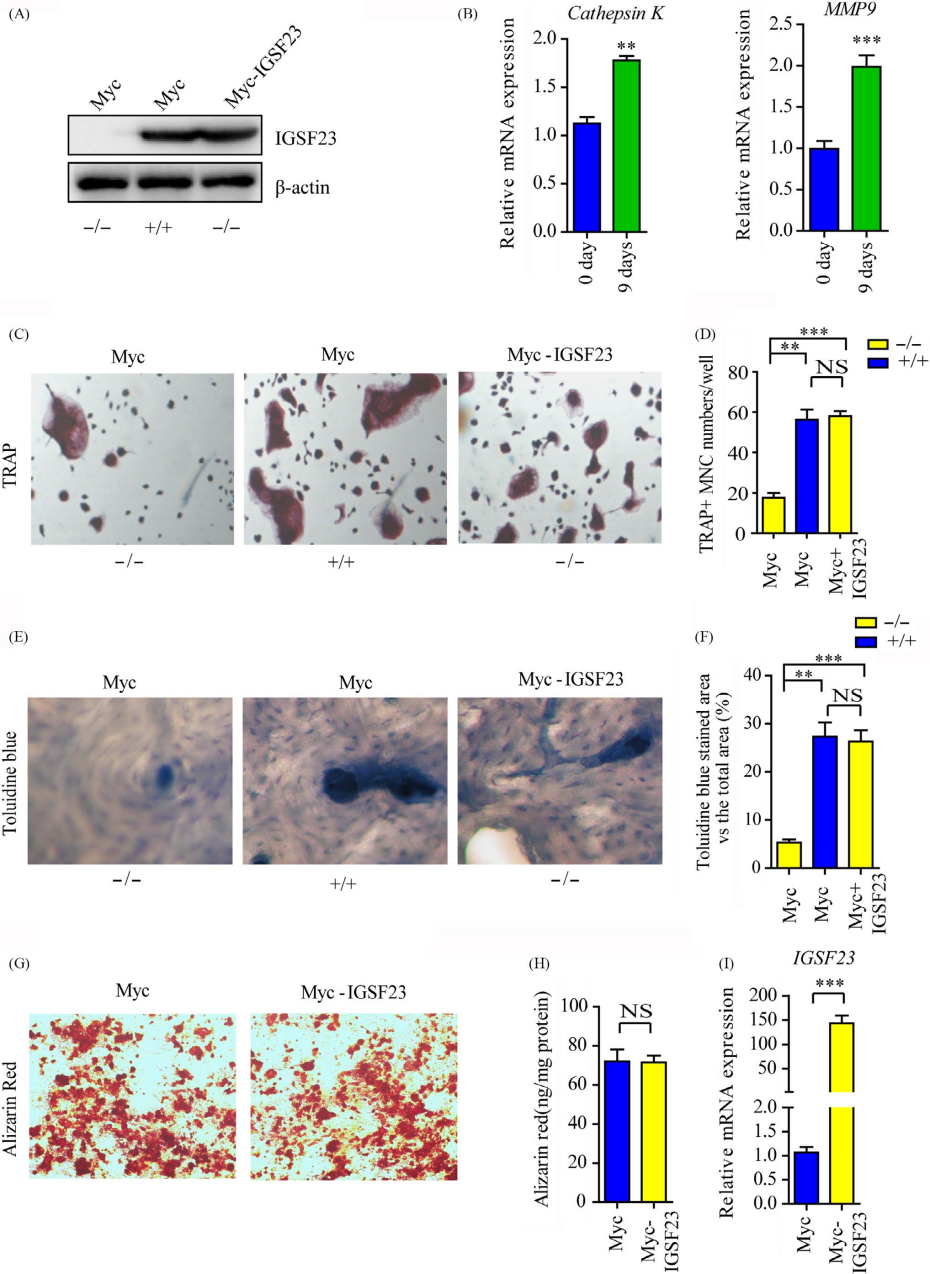
PBMCs from the proband (II:1; −/−) and her older sister (II:2; +/+) were seeded into 24‐well plates and induced by M‐CSF/RANKL along with transfected with myc‐IGSF23 or empty vector. A, PBMCs were collected for Western blot analysis incubated with IGSF23 antibody. B, RT‐qPCR analysed the mRNA levels of Cathepsin K and MMP9. C, Osteoclasts were imaged and indicated by the arrows (200×). D, The numbers of osteoclast was counted. E, The bone resorption was analysed by toluidine blue staining. F, The percentage bone resorption pit areas were measured. G, Osteoblast cells were transfected with myc‐IGSF23 or empty vector and induced osteogenic differentiation and stained with Alizarin Red staining. H, The matrix mineralization in osteoblasts transfected with myc‐IGSF23 or empty vector was analysed. I, The osteoblasts transfected with myc‐IGSF23 or empty vector and were collected for RT‐qPCR analysis. Three replicates were performed for RT‐qPCR and Western blotting analysis. **P < .01, ***P < .001. NS = not significant

To further demonstrate the effect of IGSF23 on osteoclastogenesis, PBMCs from the proband (II:1; −/−) and her older sister (II:2; +/+) were seeded into 24‐well plates and transfected with myc‐IGSF23 plasmid or myc empty vector respectively, along with incubated M‐CSF and RANKL for 7 days. Western blot analysis showed that IGSF23 protein was expressed in IGSF23^+/+^ PBMCs induced by M‐CSF/ RANKL, but not in IGSF23^−/−^. Overexpression of myc‐IGSF23 plasmid rescued IGSF23 protein expression in IGSF23^−/−^ PBMCs (Figure 5A). The mRNA levels of Cathepsin K and MMP9 were also increased in PBMCs after incubated with M‐CSF and RANKL (Figure 5B). TRAP staining showed that overexpression of IGSF23 rescued the ability of IGSF23^−/−^ PBMCs differentiate into osteoclasts (TRAP^+^) in the present of M‐CSF/RANKL (Figure 5C,D). Toluidine blue staining showed that the percentage bone resorption pit areas was also lower in PBMCs from the proband (II:1, IGSF23^−/−^) than her older sisters II:2 (IGSF23^+/+^), but the result was reversed by overexpression of IGSF23 in the proband (II:1, IGSF23^−/−^) (Figure 5E,F). Similarly, overexpression of IGSF23 does not affect osteogenesis of osteoblast (Figure 5G‐I). Altogether, these data indicated that IGSF23 promotes M‐CSF/RANKL‐induced osteoclastogenesis of PBMCs, but does not affect osteogenesis.

### 3.5 Silencing of IGSF23 reversed the bone loss in OVX mice

**Figure 6:**
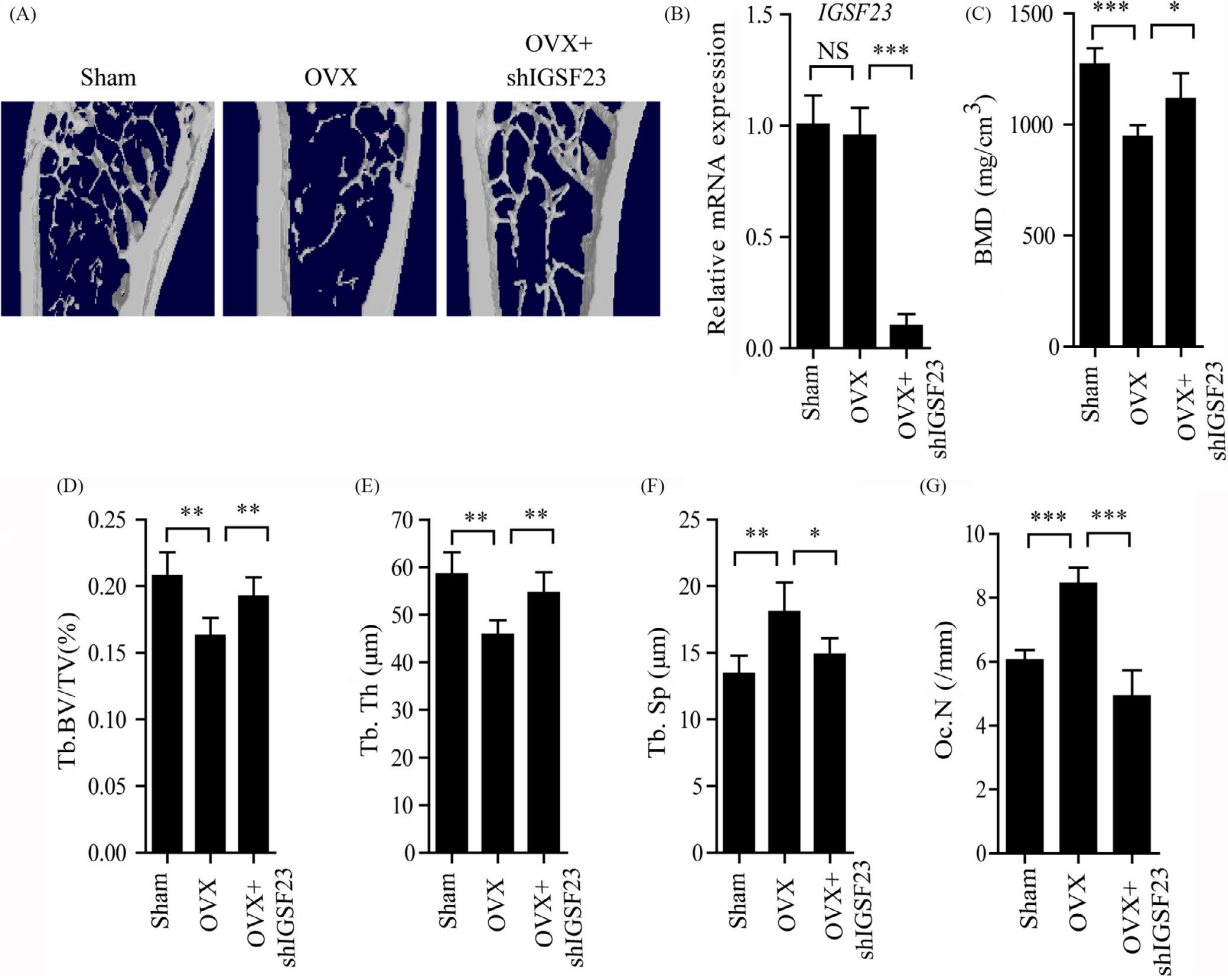
Injection of AAV‐shIGSF23 into bone marrow reversed the bone loss in OVX mice. AAV‐shIGSF23 was injected into OVX mice via periosteal twice per month for 3 months (N = 6/group). A, The effect of silencing of IGSF23 in OVX mice by microCT examination. B, RT‐qPCR analysis of the expression of IGSF23 in bone. C‐F, Quantitative μCT analysis of BMD, bone mineral density (g/cm^2^) (C); BV/TV, bone volume/ tissue volume (%) (D); Tb.Th, trabecular thickness (mm) (E) and Tb.Sp, trabecular spacing (mm) (F). G, Quantitative analysis of the number of osteoclasts on the bone surface. Data are shown as the mean ± SD, *P < .05, **P < .01, ***P < .001

Osteoporosis is caused by bone resorption that exceeds bone formation.^34^, ^35^, ^36^ Considering that the mutation of IGSF23 inhibited the osteoclast differentiation, which indicates that inhibition of IGSF23 may be regarded as a putative strategy for treating osteoporosis. To investigate the therapeutic effects of silencing IGSF23 in osteoclast differentiation, adeno‐associated virus (AAV)‐shIGSF23 was injected into the femoral bone marrow cavity of OVX mice twice per month for 3 months (n = 6). The result showed that intra‐bone marrow injection of AAV‐shIGSF23 significantly decreased the mRNA levels of IGSF23 by RT‐qPCR analysis (Figure 6B). Moreover, silencing of IGSF23 increased the bone density, trabecular bone volume per tissue volume, trabecular thickness and showed lower trabecular separation compared with vehicle‐treated mice (Figure 6A,C‐F). Furthermore, the number of osteoclasts on the bone surface was much lesser in mice treated with AAV‐shIGSF23 relative to controls (Figure 6G). These results revealed that silencing of IGSF23 inhibited the formation of osteoclast and reversed the bone loss in OVX mice.

### 3.6 IGSF23 promotes osteoclasts differentiation of PBMCs via MAPK signalling pathways

**Figure 7:**
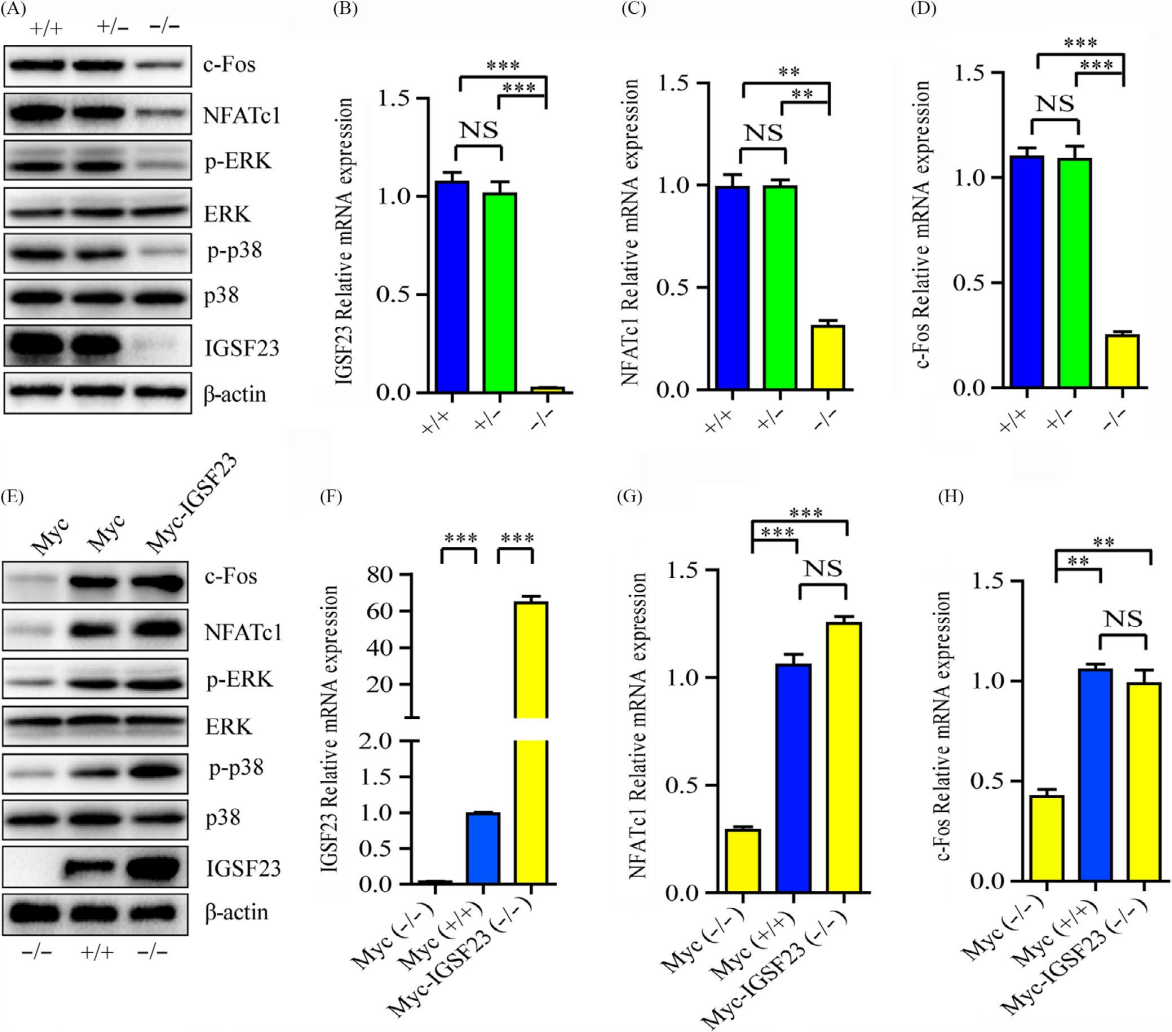
PBMCs from the proband (II:1; −/−) and her two older sisters (II:2; +/+ and II:3; +/−) were seeded into 24‐well plates and incubated with M‐CSF and RANKL for 7 days. A, PBMCs were collected for Western blot analysis incubated with c‐Fos, NFATc1, p‐ERK, ERK, p‐p38, p38, IGSF23 and β‐actin antibodies. B‐D, PBMCs were collected for qPCR analysis. E, PBMCs from the proband (II:1; −/−) and her older sister (II:2; +/+) and transfected with myc‐IGSF23 or empty vector, and then cultured with M‐CSF and RANKL for 7 days. PBMCs were collected for Western blot analysis incubated with c‐Fos, NFATc1, p‐ERK, ERK, p‐p38, p38, IGSF23 and β‐actin antibodies. F‐H, PBMCs were collected for qPCR analysis. Three replicates were performed for RT‐qPCR and Western blotting analysis. **P < .01, ***P < .001. NS = not significant

Previous studies demonstrated that RANK signalling pathways are crucial for RANKL‐stimulated osteoclast differentiation.^11^ We next examined whether IGSF23 promotes osteoclasts differentiation of PBMCs via activating RANK signalling pathways. PBMCs from the proband (II:1; −/−) and her two older sisters (II:2; +/+ and II:3; +/−) were induced by M‐CSF and RANKL for 7 days. Western blot analysis showed that the protein expression levels of p‐ERK1/2 and p‐p38 in IGSF23^−/−^ PBMCs were significantly decreased than in IGSF23^+/+^ and IGSF23^+/−^ PBMCs (Figure 7A). Overexpression of IGSF23 rescued the protein expression levels of p‐ERK1/2 and p‐p38 in IGSF23^−/−^ PBMCs compared to the control group (Figure 7E). It is a fact that c‐Fos and NFATc1 are regarded as important transcription factors for osteoclast differentiation.^37^ Next, we examined the effect of IGSF23 on the expression of c‑Fos and NFATc1. The Western blotting and qRT‐PCR results showed that the protein expression levels and mRNA levels of c‐Fos and NFATc1 were significantly decreased in IGSF23^−/−^ PBMCs than in IGSF23^+/+^ and IGSF23^+/−^ PBMCs (Figure 7A‐D), whereas overexpression of IGSF23 reversed the result in IGSF23^−/−^ PBMCs compared to the IGSF23+/+ group (Figure 7E‐H). These results suggest that IGSF23 promoted osteoclast differentiation of PBMCs by activating mitogen‐activated protein kinase (MAPK) signalling pathways.

## 4 DISCUSSION

This study has revealed for the first time the role of human IGSF23 in osteoclastogenesis and demonstrated the involvement of a nonsense mutation in IGSF23 gene in the development of human autosomal recessive osteopetrosis.

Osteopetrosis is regarded as a kind of rare inherited skeleton disease and could be fatal in infancy.^7^, ^38^ In recent years, some symptoms of osteopetrosis including anaemia, pancytopenia, obstruction of bone marrow cavity, hepatosplenomegaly, hearing loss, visual impairment and facial palsy are reported.^39^ However, in this family, the osteopetrosis subjects did not present symptoms with features of the autosomal recessive form. More notably, in spite of existing evidence from previous studies that sclerotic bones of osteopetrosis subjects are brittle and prone to fracture, the subjects in this study had no history of bone fracture. Previous studies showed the genes mutation of TCIRG1, CLCN7, LRP5, IGSF23, OSTM1, CAII, PLEKHM1, TNFSF11, TNFRSF11A, CTSK, IKBKG and ITGB3 have been shown to be associated with osteopetrosis or osteopetrosis‐like phenotypes.^10^ However, none of these mutations was detected in this study, which indicates a novel gene mutation may emerge. In our study, we found a new mutation of IGSF23, which promotes osteoclast differentiation of PBMCs via activating MAPK signalling pathways. The IGSF23 gene consists of four coding exons and spanned about 23.1 kb on chromosome 19q13.31. It encodes a protein consisting of 192 amino acids and contains an immunoglobulin‐like (IG) domain and a putative transmembrane domain in the C‐terminal region. Interestingly, it is high conserved in chimpanzee, dog, cow, mouse and rat (Figure 3B). In our study, we detected a homozygous mutation (c.295C>T) in the IGSF23 coding region from two osteopetrosis samples by using genome‐wide scan and fine mapping study analysis. But, the mutation was not detected in 180 healthy control samples. Heterozygous mutations were detected in the parents and the sister of the proband although none of them suffered from osteopetrosis. These data suggest that the homozygous mutation (c.295C>T) of IGSF23 was strongly associated with osteopetrosis. It is indeed that the homozygous mutation (c.295C>T) of IGSF23 leads to form a stop codon which codes a mutant form of IGSF23 protein losing the immunoglobulin‐like domain and the whole transmembrane domain (Figure 3C). The deletion mutation of IGSF23 may be lead to lose its function compared with the full‐length protein.

Following, the role of IGSF23 in the differentiation of osteoclasts in vitro was then investigated. QRT‐PCR analysis revealed that the IGSF23 mainly expresses in osteoclasts, bone and small intestine. Besides, the mRNA level and protein expression of IGSF23 was increased during M‐CSF/RANKL‐induced differentiation of PBMCs. This suggests IGSF23 involves in osteoclast differentiation. Bone biopsy of the proband showed the number of mature osteoclasts was much less than her old sister (II:2). Moreover, the ability of osteoclastic differentiation of PBMCs from IGSF23^−/−^ proband is weak compared to her two older sisters. Furthermore, overexpression of IGSF23 rescued the osteoclastogenesis of PBMCs from IGSF23^−/−^ proband. These results indicated that the mutation of IGSF23 results in the osteopetrosis phenotype, and suggested that IGSF23 is important for M‐CSF/RANKL‐induced osteoclastogenesis.

The mechanism of IGSF23 involvement in osteoclast differentiation was then investigated. Osteoclast differentiation is depended on the activation of RANK which binds to the RANKL.^40^ And then, RANK‐RANKL stimulates some transcription factors expression for osteoclast differentiation by activating MAPK or NF‐κB pathway.^40^, ^41^, ^42^ Among them, c‐Fos and NFATc1 are regarded as critical transcription factors for osteoclastogenesis.^43^, ^44^ Previous studies showed that c‐Fos and NFATc1 knockout mice develop severe osteopetrotic phenotype with complete deficiency of osteoclasts.^43^ Our results in this study demonstrate that PBMCs from the proband (IGSF23 mutation) lost the osteoclastogenic potential, because the mutation of IGSF23 lead to decrease the phosphorylation of ERK1/2 and p38 and then inhibit the expression of c‐Fos and NFATc1.

In conclusion, this study identified a novel gene mutation of IGSF23, which caused a rare form of autosomal recessive osteopetrosis. IGSF23 is selectively expressed in osteoclasts and promotes M‐CSF and RANKL‐stimulated osteoclast differentiation via activating MAPK signalling pathways. Therefore, IGSF23 may be regarded as a new target for osteoporosis.

## Data Availability

The data sets used and/or analysed during the current study are available from the corresponding author on reasonable request.
