# Supplementary material for: Osteoclastogenesis inhibition by mutated IGSF23 results in human osteopetrosis
Source: Cell Prolif. 2019 Sep 27;52(6):e12693. doi: 10.1111/cpr.12693 (PMC6869366; doi:10.1111/cpr.12693)
Supplement: Supplementary file 1 [file CPR-52-e12693-s001.docx]

**Supplementary Material**

Table S1 Primer pairs in this study

| Gene | Sequence (5′-3′) |
| --- | --- |
| IGSF23_exon 1 _F | 5'-CCATTCCTTGCCTTTGATGT-3' |
| IGSF23_exon 1 _R | 5'-TGTTCTGTCCAGGGACTGTG-3' |
| IGSF23_exon 2 _F | 5'-ACTCCACTCTTGTGGCCTTG-3' |
| IGSF23_exon 2 _R | 5'-AAAGGGTCTCACAGGGGACT-3' |
| IGSF23_exon 3 _F | 5'-CAGATGGCTCCTCCAATCTC-3' |
| IGSF23_exon 3 _R | 5'-TTCCTTGTTGAGCCAGCTCT-3' |
| IGSF23_exon 4 _F | 5'-CGATGATTCTTATTCTCTCTCTCTCTC-3' |
| IGSF23_exon 4 _R | 5'-GAACCAGGAGGGAGGAAATC-3 ' |
